# Mechanisms of NOS1AP action on NMDA receptor-nNOS signaling

**DOI:** 10.3389/fncel.2014.00252

**Published:** 2014-08-27

**Authors:** Michael J. Courtney, Li-Li Li, Yvonne Y. Lai

**Affiliations:** ^1^Molecular Signalling Laboratory, Department of Neurobiology, A. I. Virtanen Institute, University of Eastern FinlandKuopio, Finland; ^2^Turku Centre for Biotechnology, Abo Akademi University and University of TurkuTurku, Finland; ^3^Jack Gill Center for Biomolecular Science, Department Psychological and Brain Sciences, Indiana UniversityBloomington, IN, USA

**Keywords:** NOS1AP, nNOS, NMDA receptor, PSD95, PDZ, nitric oxide, excitotoxicity, schizophrenia

## Abstract

NMDA receptors (NMDAR) are glutamate-gated calcium channels that play pivotal roles in fundamental aspects of neuronal function. Dysregulated receptor function contributes to many disorders. Recruitment by NMDARs of calcium-dependent enzyme nNOS via PSD95 is seen as a key contributor to neuronal dysfunction. nNOS adaptor protein (NOS1AP), originally described as a competitor of PSD95:nNOS interaction, is regarded an inhibitor of NMDAR-driven nNOS function. In conditions of NMDAR hyperactivity such as excitotoxicity, one expects NOS1AP to be neuroprotective. Conditions of NMDAR hypoactivity, as thought to occur in schizophrenia, might be exacerbated by NOS1AP. Indeed GWAS have implicated NOS1AP and nNOS in schizophrenia. Several studies now indicate NOS1AP can mediate rather than inhibit NMDAR/nNOS-dependent responses, including excitotoxic signaling. Yet the concept of NOS1AP as an inhibitor of nNOS predominates in studies of human disease genetics. Here we review the experimental evidence to evaluate this apparent controversy, consider whether the known functions of NOS1AP might defend neurons against NMDAR dysregulation and highlight specific areas for future investigation to shed light on the functions of this adaptor protein.

## NOS1AP and disease

The *nos1ap* gene is linked to diseases including schizophrenia (Brzustowicz et al., 2004; Xu et al., 2005), post-traumatic stress disorder and depression (Lawford et al., 2013), autism (Delorme et al., 2010), sudden cardiac death and long QT syndromes (Newton-Cheh et al., 2009; Kapoor et al., 2014) and diabetes (Becker et al., 2008). The gene encodes NOS1 Adaptor Protein (NOS1AP), initially called carboxy-terminal PSD95-Dlg-ZO1 (PDZ) ligand of nNOS (CAPON; Jaffrey et al., 1998) because it binds the N-terminal PDZ-containing region of nNOS. The protein contains a C-terminal class II PDZ-motif (ψxψ-COOH) and an N-terminal phosphotyrosine binding (PTB) domain but no other recognizable domains. NOS1AP was originally described as an inhibitor of NMDA receptor (NMDAR)-driven nNOS actions because in cell-free assays it reduced interaction between nNOS and PSD95, the protein recruiting nNOS to NMDAR (Jaffrey et al., 1998). In contrast, later studies suggested NOS1AP *mediates* NMDAR-driven actions of nNOS (Fang et al., 2000; Cheah et al., 2006; Li et al., 2013). Nevertheless, in human disease studies, NOS1AP continues to be described as an inhibitor of nNOS (Eastwood, 2005; Xu et al., 2005; Qin et al., 2010; Weber et al., 2014). Despite this disparity of views, a consensus is emerging that nNOS:NOS1AP interaction is a potential drug target for neurological and cardiovascular disorders (Li et al., 2013; Kapoor et al., 2014; Weber et al., 2014). The rapidly accumulating reports linking NOS1AP to psychiatric and cardiovascular diseases increase focus on the druggability of NOS1AP functions. We therefore believe it is timely to discuss models for NOS1AP regulation of NMDAR-driven nNOS signaling.

## NMDAR-driven nNOS signaling and the involvement of PSD95

To address the possible significance of NOS1AP regulation of NMDAR-driven nNOS functions, we briefly overview the relationship of nNOS to NMDAR signaling. NMDARs regulate neuronal development, survival and physiology but also contribute to neuronal dysfunction and disease, from stroke and neurodegenerative disorders to psychiatric disorders and chronic pain (Kemp and McKernan, 2002; Salter and Pitcher, 2012; Citrome, 2014). NMDAR signaling through nNOS contributes to excitotoxicity and thus lesions in both stroke and neurodegenerative diseases (Aarts et al., 2002; Lai et al., 2014), while atrophy caused by excitotoxicity may contribute to depression (Rajashekaran et al., 2013; Vu and Aizenstein, 2013; Stein et al., 2014). NMDAR signaling attracts interest as a potential therapeutic target because inhibitors of steps in the pathway from NMDAR to nNOS are effective in models of many disorders (Kemp and McKernan, 2002; Hashimoto, 2009; Doucet et al., 2012; Mellone and Gardoni, 2013; Lai et al., 2014; Mukherjee et al., 2014). However, side-effects of NMDAR antagonists have limited their clinical potential. After decades of disappointing results in clinical trials targeting the NMDAR and calcium influx, 2012 saw the first successful stroke trail targeting the signaling pathway downstream from NMDAR activation and calcium influx (Hill et al., 2012).

The interaction between NMDARs and nNOS is well understood. NMDARs gate flux of calcium as well as sodium across the plasma membrane, and sustained activation of the receptor leads to substantially increased intracellular concentrations of both ions in neurons (Courtney and Nicholls, 1992). It is calcium/calmodulin that activates nNOS. nNOS has long been recognized as a major player in disorders from excitotoxic lesions to chronic pain (Florio et al., 2009; Mukherjee et al., 2014), but catalytic inhibitors have yet to benefit patients. Perhaps they would not be tolerated given the physiological importance of nNOS and other isoforms in the heart, vasculature and other sites. Importantly, calcium influx alone does not strongly activate nNOS; PSD95 is necessary to efficiently couple NMDAR-gated calcium influx to nNOS activation (Christopherson et al., 1999; Aarts et al., 2002; Ishii et al., 2006). The ternary complex assembling NMDAR, PSD95 (or related MAGUKs) and nNOS was characterized over 15 years ago (Christopherson et al., 1999; recently reviewed in Doucet et al., 2012) and has become particular interesting for development of therapeutic agents. Protein interactions have recently emerged as viable druggable targets, even in the most challenging conditions (Blazer and Neubig, 2009; Hill et al., 2012), and may provide alternative more selective approaches than inhibiting nNOS or NMDAR directly. Clearly, understanding interactions between proteins is essential for optimal development of novel drug leads that target protein-protein interactions, which could contribute to new treatments for clinically relevant conditions such as stroke, schizophrenia, chronic pain and cardiovascular diseases.

Key events downstream of NMDAR-evoked nNOS activation of relevance to neuronal disorders have remained rather nebulous. Substantial evidence supports a role for nNOS in excitotoxicity, but discrepancies exist. In hippocampal slices for example, μM of exogenous NO had no deleterious effects while excitotoxic stress was found to only generate 1000-fold lower concentrations of NO as measured by *in situ* assay (Keynes et al., 2004). NO generated by nNOS may reach high concentrations in the immediate vicinity of the active NMDAR/nNOS complex, but even a micrometer from the complex, diffusion and metabolism would considerably lower [NO] (Keynes et al., 2004; Philippides et al., 2005). The average cellular [NO] may therefore appear below the threshold for regulation of most NO targets. In such a scenario, interactions of nNOS with target proteins become critical determinants of downstream actions of the limited amounts of NO generated (Li et al., 2013).

## Signaling proteins downstream of nNOS

Candidate downstream mediators of nNOS include p38MAPK and JNKs. Both are involved in disorders including neurodegeneration, cerebral ischemia and chronic pain (reviewed in Ji et al., 2009; Lai et al., 2014). Activation of p38MAPK in neurons is induced by NO, generated either from donors (Ghatan et al., 2000) or NMDAR-stimulation (Cao et al., 2005; Soriano et al., 2008; Li et al., 2013). JNK activation, not an obligate component of excitotoxicity (Cao et al., 2004; note isoforms may be regulated in opposite ways, Brecht et al., 2005), may involve distinct pathways (Soriano et al., 2008). NO can even inhibit JNK via S-nitrosylation (Park et al., 2000). NMDAR-driven nNOS activation may be specifically coupled to p38MAPK responses (Cao et al., 2005; Soriano et al., 2008) via NOS1AP (Li et al., 2013). NMDAR stimulation recruits NOS1AP to nNOS in neuronal cells and siRNAs targeting NOS1AP inhibits excitotoxic death and p38MAPK activation (Li et al., 2013). Moreover, a peptide designed to selectively disrupt the binding of nNOS to NOS1AP inhibits both p38MAPK and cell death/lesions in *ex vivo* and *in vivo* excitotoxicity models. Cell death was also prevented by overexpression of the nNOS-PDZ binding pocket without reducing calcium or nitric oxide responses. This suggests that competition for NOS1AP:nNOS interaction can be achieved by targeting either one or other side of the interaction i.e., providing either nNOS ligand peptide or the free nNOS ligand-binding domain, thereby inhibiting events downstream of PSD95:nNOS interaction. The observation that NOS1AP contributes to nNOS-dependent cell death is significant as it potentially expands the range of druggable targets in multiple disorders. However, this conclusion appears controversial as NOS1AP was originally described as a competitive inhibitor of nNOS function, and continues to be referred to as such, even though several other studies suggest that NOS1AP in fact mediates actions of NMDAR-driven nNOS (see Section NOS1AP and disease). To address this controversy, here we review how NOS1AP might contribute to NMDAR signaling pathways, taking into account the structural motifs critical for interactions between these three proteins. We discuss previously proposed models and develop for consideration an additional model of nNOS/PSD95/NOS1AP function that is more consistent with experimental findings.

## Regulation of nNOS interactions

### The exclusion model

**Figure 1:**
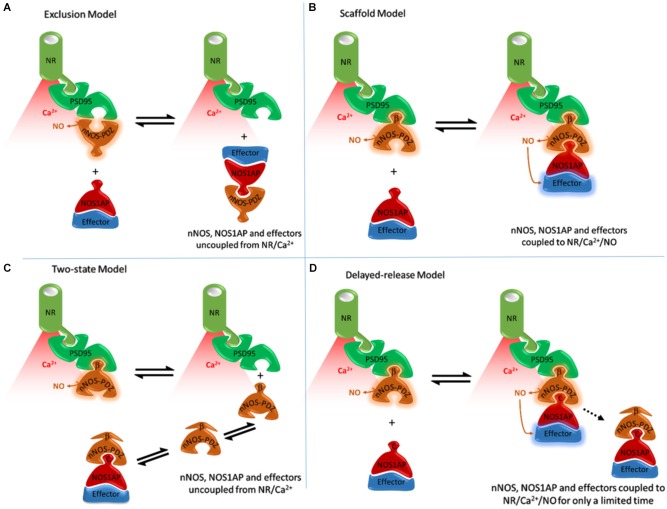
**Alternative models of NOS1AP interaction with nNOS and their anticipated consequences to exposure of NOS1AP effector to nitric oxide**. **(A)** The Exclusion Model, based on Jaffrey et al. (1998) and Eastwood (2005). Binding of PSD95 to nNOS excludes binding of NOS1AP by PDZ-PDZ interaction and direct competition and vice versa. A PDZ-PDZ interaction was originally envisioned (Jaffrey et al., 1998), but this is consistent neither with structural nor functional data. Coupling of nNOS to NMDAR/Ca2+-influx is important for activation (Aarts et al., 2002; Ishii et al., 2006). Therefore in all schemes nNOS, when coupled to NMDAR (via PSD95) is shown producing NO (active), whereas nNOS displaced from NMDAR/Ca2+-influx (red shading) is depicted without NO production. In this model the nNOS/NOS1AP complexes with effectors such as DexRas would not be directly localized to the receptor and calcium influx-associated NO produced. **(B)** The Scaffold Model, based on Christopherson et al. (1999) and Li et al. (2013). Binding of nNOS β-finger to PSD95 facilitates an extended complex incorporating NOS1AP (or other ligands with C-terminal motifs). This model places nNOS close to the source of calcium influx, and NOS1AP effectors close to NO produced. This is consistent with NOS1AP mediating actions of NMDAR activated nNOS (Fang et al., 2000; Cheah et al., 2006; Li et al., 2013). But it is not consistent with cell-free experiments in which NOS1AP competes with PSD95 for binding nNOS (Jaffrey et al., 1998). **(C)** The Two-state model. The extended PDZ domain of nNOS is proposed to exist in two conformational states. One can bind PSD95 not NOS1AP, the other NOS1AP not PSD95. This could explain competition between PSD95 and NOS1AP. This model, however, places the nNOS-NOS1AP complex at a distance from the NMDA receptor, limiting activation of nNOS in the nNOS-NOS1AP complex. This is not consistent with NOS1AP mediating NMDAR/nNOS-dependent pathways (Fang et al., 2000; Cheah et al., 2006; Li et al., 2013). **(D)** The Delayed-release model. Here NOS1AP can interact with the unoccupied PDZ pocket seen in Figure 2, allowing the coupling of NMDAR/nNOS signaling to NOS1AP dependent pathways. But undefined mechanisms gradually lead to the loss of PSD95 binding by the beta-finger, presumably via conformational changes, resulting in delayed dissociation of the nNOS-NOS1AP complex from the receptor. In this model, the nNOS/NOS1AP effector complex is localized with the receptor and associated calcium influx for a limited time only. This model potentially explains the apparently conflicting data on NOS1AP function.

The N-terminal region of nNOS that binds both PSD95 and NOS1AP contains a PDZ domain followed by additional sequences. PDZ domains are conserved 90 amino acid regions typically recognizing short C-terminal peptide motifs (Doyle et al., 1996). Some PDZ domains, like in nNOS, have flanking sequences or extensions which may confer additional properties, but these are not part of the core PDZ domain (Wang et al., 2010). The canonical PDZ interaction involves the docking of the last three residues at the extreme C-terminus of the peptide ligand into a “binding pocket” formed between β-sheet 2 and α-helix 2 of the PDZ domain (Doyle et al., 1996; Harris and Lim, 2001). The interaction between nNOS and PSD95 is unlike this canonical PDZ interaction. The initial finding that the N-terminal region of nNOS (containing a PDZ domain) interacts with a PDZ domain of PSD95 was taken to indicate that core PDZ domains could form a dimer, instead of merely binding C-terminal peptides. The only known interaction site at the time was the ligand-binding pocket, so PDZ dimerization was thought to exclude binding of C-terminal ligands (Jaffrey et al., 1998). We refer to this original concept as the *Exclusion Model* (Figure 1A). Consistent with this, recombinant NOS1AP C-terminus inhibits the interactions between nNOS and PSD95 interaction, both by GST-PSD95 pull-down of nNOS from 293T cell lysates and by co-immunoprecipitation of PSD95 with nNOS from 293T cells overexpressing nNOS, PSD95 and NOS1AP (Jaffrey et al., 1998). Thus nNOS:PSD95 interaction was seen as a dimerization of core PDZ domains, and nNOS-PDZ ligands like NOS1AP would compete with PSD95 for interaction i.e., the dimerization occludes both ligand-binding pockets (Figure 1A).

**Figure 2:**
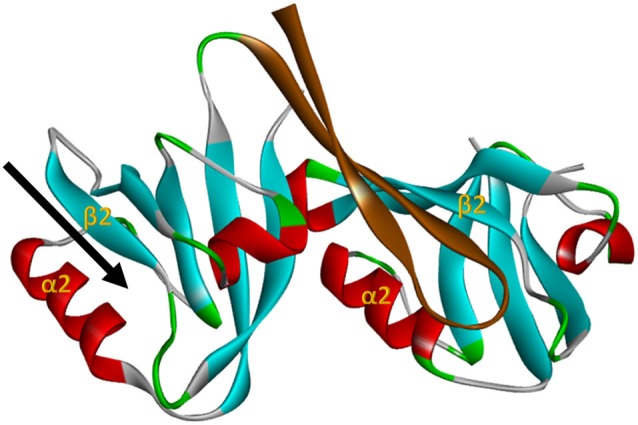
**The nNOS:PSD95 interaction.** Projection of crystal structure 1QAV.pdb (Hillier et al., 1999) of the nNOS extended PDZ domain (left) docking with a classI PDZ domain (right, from syntrophin in this case). The parallel β-sheet 2/α-helix 2 regions that form the ligand binding pockets of the PDZ domains are labelled in each domain. The β finger of the nNOS extended PDZ that docks in the classI PDZ domain is shown in brown, revealing that the PDZ ligand binding pocket of nNOS (arrow, left) remains unoccupied. This projection was generated with Accelrys Discovery Studio.

### The scaffold model

Subsequent structural studies clearly showed the core PDZ domain of nNOS is *not* itself the binding partner of PSD95-type PDZ domains (Hillier et al., 1999; Tochio et al., 1999, 2000; Figure 2), directly contradicting the exclusion model. The core PDZ domains that contain ligand-binding pockets do not interact (Christopherson et al., 1999). Instead, the flanking motif outside the core nNOS PDZ domain, known as the β-finger, mediates the interaction with PSD95-type PDZ domains. Thus nNOS:PSD95 is a heterodimer in which each protein contains one or more PDZ domains (Figure 2). Although not the PDZ dimer originally envisaged (Figure 1A), this dimeric interaction is still not a canonical PDZ domain:C-terminal peptide interaction and is referred to as a non-canonical PDZ interaction (Lenfant et al., 2010). Core PDZ domains in some cases form dimers by domain-swapping (Chen et al., 2008; reviewed in Lee and Zheng, 2010), but dimer formation does not occlude ligand binding pockets. The revised model (Figure 1B) allows the intriguing possibility that the ligand-binding pocket of this class III-type PDZ domain remains available for further recruitment of its own specific targets (Christopherson et al., 1999; Figures 2, 1B, *Scaffold Model*).

NOS1AP may be one such target. In contrast to nNOS:PSD95 interaction, the core PDZ domain of nNOS (residues 1–100) *is* sufficient to interact with NOS1AP. Furthermore, C-terminal residues of NOS1AP are required for interaction (Jaffrey et al., 1998). On this basis, nNOS:NOS1AP binding is a canonical PDZ interaction. The scaffold model is consistent with this and with the original data describing the interaction between PSD95 and the N-terminal region of nNOS (Brenman et al., 1996). However it conflicts with the observed inhibition of nNOS:PSD95 interaction by NOS1AP C-terminus (Jaffrey et al., 1998) and potentially other ligands as envisaged in the exclusion model.

### The two-state model

Although the behavior of NOS1AP in cell-free systems is inconsistent with the scaffold model, peptide ligands for nNOS-PDZ terminating with the motif G(D/E) × V did not inhibit nNOS:PSD95 interaction and YAGQWGESV peptide co-precipitated in a ternary complex with nNOS and PSD95-PDZ2 (Christopherson et al., 1999; Li et al., 2013). Notably, inhibition of PSD95:nNOS interaction by NOS1AP has been assumed to be competitive, but this was not demonstrated (Jaffrey et al., 1998). Inhibition could instead be non-competitive or allosteric. If the C-terminal peptide of NOS1AP would dock in the nNOS-PDZ binding pocket in a manner distinct from non-inhibitory peptides mentioned above, stabilizing an allosteric change of conformation that precludes PSD95 from binding the beta-finger of nNOS, this could explain the inhibition of PSD95:nNOS interaction by NOS1AP. We call this the *Two-State Model* (Figure 1C).

However, the Zhang lab specifically considered this in their NMR spectroscopy study but found no evidence to support it. They noted that NOS1AP peptide does *not* compete with PSD95 and concluded NOS1AP was “unlikely to compete with PSD-95 for nNOS” (Tochio et al., 1999). The two-state model predicts that events increasing nNOS-NOS1AP interaction should be accompanied by reduced nNOS-PSD95 interaction. Acquisition of a distinct conformation of nNOS driven by NMDAR activity might cause release from PSD95, and explain the increased nNOS:NOS1AP interaction and consequential downstream signaling reported (Li et al., 2013). However, PSD95-nNOS co-immunoprecipitation *increases* upon excitotoxic stimulation in neuronal cultures, hippocampal slices and ischemic brain (Zhou et al., 2010). Furthermore, the laboratory that discovered the inhibition of nNOS:PSD95 interaction by NOS1AP later proposed NOS1AP *mediates* NMDAR-driven nitrosylation and activation of NOS1AP ligand DexRas (Fang et al., 2000), which participates in NMDA-evoked activation of iron uptake (Cheah et al., 2006). The two-state model (Figure 1C) is not consistent with this, as nNOS/NOS1AP complexes do not interact with the NMDAR/PSD95 complex gating calcium influx (Figure 1C) and yet proximity of nNOS with NMDAR is considered important for nNOS activation and downstream functions (Cao et al., 2005; Ishii et al., 2006; Soriano et al., 2008; Li et al., 2013). Indeed, so important that inhibiting this interaction is a valid and successful strategy for neuroprotection from NMDAR/nNOS-dependent toxicity (Aarts et al., 2002; Hill et al., 2012).

### The delayed-release model

Here we formulate an alternative model which proposes that competition with PSD95 observed in cell-free conditions (that utilize prolonged incubations) *does* occur in intact systems but only after a delay. The PDZ ligand-binding pocket of nNOS is clearly separate from the beta-finger ligand of nNOS that binds PSD95 (Figure 2), and docking of ligand in the pocket itself was reported *not* to affect the beta finger structure (Tochio et al., 2000). Thus any competition between NOS1AP and PSD95 necessitates secondary allosteric alteration of the nNOS:PSD95 interface. This may not necessarily take place instantaneously. Signaling downstream of nNOS, such as p38MAPK activation, shows transient activation in stimulated cells (Cao et al., 2005) and therefore possesses an obligate inhibitory or normalization phase. Perhaps NOS1AP, in the cellular context, acts as an inhibitor of nNOS:PSD95 interaction only after a delay, to shut down signaling once it has been activated. This delayed-release model, shown in Figure 1D, might explain the coupling of NOS1AP effectors to NMDAR-driven nNOS activation and the opportunities to inhibit signaling by competition at the nNOS-PDZ domain. This may be the most attractive model as it can reconcile most if not all apparently conflicting experimental observations. Currently however, no experimental evidence directly supports such a sequence of events nor are there any known mechanisms to explain how this might occur.

## NOS1AP—inhibitor or mediator?

NOS1AP was regarded as an *inhibitor* of nNOS function (Jaffrey et al., 1998), particularly when discussing the possible relevance of NOS1AP to human disease (Xu et al., 2005; Qin et al., 2010; Weber et al., 2014). However, functional studies suggest NOS1AP is a *mediator* of nNOS signaling and contributor to NMDAR/nNOS-dependent regulation of neuronal functions (Fang et al., 2000; Cheah et al., 2006). Decreased expression of NOS1AP by RNAi or peptide competitors of nNOS:NOS1AP interaction inhibited NMDAR/nNOS-evoked events in neurons (Li et al., 2013). Notably, NOS1AP competition of nNOS:PSD95 interaction has been demonstrated entirely in cell-free experiments and no actual evidence of *functional* inhibition of nNOS has actually been reported (Jaffrey et al., 1998). Importantly, NOS1AP does *not* directly inhibit the enzyme activity of nNOS (Jaffrey et al., 1998). Inhibition of NMDAR-evoked nNOS activity has merely been inferred from its ability to inhibit PSD95:nNOS interaction in cell-free systems. In contrast, evidence for NOS1AP as a facilitator of nNOS-mediated NMDAR signaling to activation of DexRas, iron transport, p38MAPK and neurodegeneration derives from intact cells and animal models (Fang et al., 2000; Cheah et al., 2006; Li et al., 2013). Similarly, the effects of NOS1AP on neurite architecture are reduced by nNOS inhibitor L-NAME (Carrel et al., 2009). This supports the role of NOS1AP as a positive mediator of nNOS signaling. Apparent discrepancies may arise from differences between cell-free and more physiological systems used. Additional components or dynamic properties (like the proposed delayed release, Figure 1D) of intact neuronal environments absent from those cell-free binding experiments showing inhibition by NOS1AP of nNOS:PSD95 interaction are among the possible contributing factors. The evidence for NOS1AP mediating NMDAR/nNOS signaling in intact systems is therefore more convincing than evidence to the contrary.

## Future perspectives for NOS1AP research

The potential of nNOS:NOS1AP interaction as a candidate drug target for neurological and cardiovascular disorders (Li et al., 2013; Kapoor et al., 2014; Weber et al., 2014) highlights the need to address a number of issues. Is NOS1AP interaction with nNOS more complex than assumed (e.g., Figure 1D)? How is interaction between NOS1AP and nNOS regulated? Does NOS1AP have functions independent of nNOS, for which targeting nNOS:NOS1AP interaction may be irrelevant or even potentiating? Most challenging perhaps, do functions differ under conditions of health, in response to trauma or stress, during disease? Is it more desirable to promote or disrupt NOS1AP function? Ultimately does NOS1AP act as the inhibitor originally envisioned, playing a role in self-defence of the neuron against excessive input to nNOS signaling?

Pre-conditioning should also be considered. While p38MAPK mediates excitotoxicity, paradoxically it also facilitates the resistance to toxic insults preceded by prior sub-toxic stimuli, in brain and tissues such as heart and liver (Hausenloy and Yellon, 2006; Alchera et al., 2010; Zhao et al., 2013). As NOS1AP mediates NMDAR-evoked p38MAPK activation (Li et al., 2013), does it also contribute to pre-conditioning pathways? Should the same question be addressed for NMDAR:PSD95 and PSD95:nNOS interactions, which are considered as therapeutic targets, and for NR2B and extra-synaptic NMDARs which are much discussed as contributors to excitotoxic pathways (Hardingham and Bading, 2010) and may be preferentially linked to p38MAPK activation (Dau et al., 2014)?

Clearly more work is required to understand the impact of NOS1AP on NMDAR-driven nNOS signaling pathways, both at the molecular level as well as in a range of translational models for those diseases and conditions urgently needing new therapeutic approaches, including neurodegenerative diseases, stroke, chronic pain, as well as depression and other psychiatric conditions. Only then can we determine whether we should seek to boost endogenous functions of NOS1AP or to inhibit its function to achieve desirable therapeutic outcomes.

## Conflict of interest statement

The authors declare that the research was conducted in the absence of any commercial or financial relationships that could be construed as a potential conflict of interest.
